# A Randomized Double-Blinded, Placebo-Controlled Trial Investigating the Effect of Fish Oil Supplementation on Gene Expression Related to Insulin Action, Blood Lipids, and Inflammation in Gestational Diabetes Mellitus-Fish Oil Supplementation and Gestational Diabetes

**DOI:** 10.3390/nu10020163

**Published:** 2018-01-31

**Authors:** Mehri Jamilian, Mansooreh Samimi, Naghmeh Mirhosseini, Faraneh Afshar Ebrahimi, Esmat Aghadavod, Mohsen Taghizadeh, Zatollah Asemi

**Affiliations:** 1 Endocrinology and Metabolism Research Center, Department of Gynecology and Obstetrics, School of Medicine, Arak University of Medical Sciences, Arak 6618634683, Iran; Jamilian.mehri@gmail.com; 2 Department of Gynecology and Obstetrics, School of Medicine, Kashan University of Medical Sciences, Kashan 8715988141, Iran; dr_samimi.2007@yahoo.com (M.S.); f.afsharebrahimi@yahoo.com (F.A.E.); 3 Pure North S’Energy Foundation, Calgary, AB T2R 0C5, Canada; namirhossini@gmail.com; 4 Research Center for Biochemistry and Nutrition in Metabolic Diseases, Kashan University of Medical Sciences, Kashan 8715988141, Iran; aghadavod_m@yahoo.com (E.A.); taghizadeh.mohsen22@gmail.com (M.T.)

**Keywords:** fish oil, gene expression, insulin, lipid, inflammation, gestational diabetes

## Abstract

Gestational diabetes mellitus (GDM) is a common complication of pregnancy, and it is mostly associated with postpartum diabetes, insulin resistance, and dyslipidemia. Fish oil (omega-3) supplementation has been shown to reduce the risk of different chronic diseases such as cardiovascular disease, type 2 diabetes, and cancers, though the evidence of its impact on gestational diabetes is scarce. Our goal in this study was to determine the effect of fish oil administration on gene expression related to insulin action, blood lipids, and inflammation in women with GDM. Participants with GDM (*n* = 40), aged 18–40 years, were randomized to take either 1000 mg fish oil capsules, containing 180 mg eicosapentaenoic acid and 120 mg docosahexaenoic acid (*n* = 20), or placebo (*n* = 20) twice a day for 6 weeks. Gene expression related to insulin, lipids, and inflammation was quantified in peripheral blood mononuclear cells (PBMCs) of GDM women using Reverse Transcription Polymerase Chain Reaction (RT-PCR) method. Results of RT-PCR indicated that omega-3 supplementation upregulated gene expression of peroxisome proliferator-activated receptor gamma (PPAR-γ) (*P* = 0.04) in PBMCs of patients with GDM, compared with the placebo. In addition, gene expression of the low-density lipoprotein receptor (LDLR) (*P* < 0.001), interleukin-1 (IL-1) (*P* = 0.007), and tumor necrosis factor alpha (TNF-α) (*P* = 0.01) was downregulated in PBMCs of women with GDM, following omega-3 supplementation. No significant effect of omega-3 supplementation was indicated on gene expression of IL-8 in PBMCs of patients with GDM. Overall, fish oil supplementation for 6 weeks in women with GDM significantly improved gene expression of PPAR-γ, IL-1, and TNF-α, but not gene expression of IL-8.

## 1. Introduction

Gestational diabetes (GDM) is defined as impaired glucose metabolism and insulin resistance, presenting with high blood sugar level during pregnancy not clearly recognized as diabetes [1]. It is one of the most common complications of pregnancy [2], affecting from <1% to 28% of pregnancies worldwide, in respect of population demographics, screening tools and diagnostic criteria, and maternal lifestyle [3]. GDM increases the risk of type 2 diabetes mellitus (T2DM) and coronary heart disease (CHD) compared with healthy pregnant women [4]. Women experiencing GDM might have up to 50% risk of developing type 2 diabetes within five years after pregnancy [5]. GDM might be associated with elevated levels of inflammatory cytokines [6,7], maternal insulin concentrations leading to insulin resistance [8], and the potential of stimulating the production of inflammatory factors in placenta [9].

The fatty acid profile might be compromised in patients suffering from GDM [10], which suggests a possible relationship between fatty acids and glucose metabolism in GDM. On the other hand, epidemiological studies investigating the association between omega-3 intake and risk of GDM have been inconclusive [11,12]. Previously, we showed the beneficial effects of omega-3 intake on glucose metabolism, lipid fractions, and inflammatory factors in gestational diabetes [13,14]. With the focus on related genes expression, in a study conducted by Bassaganya-Riera et al. [15], in an animal model of inflammatory bowel disease (IBD), omega-3 intake upregulated colonic peroxisome proliferator-activated receptor gamma (PPAR-γ) expression. Moreover, the suppression of gene expression of tumor necrosis factor alpha (TNF-α) by EPA was reported in human monocytic THP-1 cells [16]. However, Mansoori et al. [17] did not find any beneficial effect of fish oil supplementation for 8 weeks, on the expression of PPAR-γ gene, in patients with T2DM.

Taking omega-3 fatty acids might improve gene expression related to insulin action, blood lipids, and inflammation by inhibiting phosphatidylinosinitol 3-kinase and protein kinase B (PKB) pathway [18], and modification of the nuclear factor kappa-light-chain-enhancer function of the activated B cells (NF-κB) pathway [19]. Data demonstrating the effects of omega-3 fatty acids supplementation on gene expression related to insulin action, blood lipids, and inflammation in GDM are scarce. We designed this study, therefore, to evaluate the effects of omega-3 fatty acids supplementation on gene expression related to insulin action, blood lipids, and inflammation in women with GDM.

## 2. Materials and Methods

### 2.1. Study Design and Participants

This study was a 6-week randomized double-blinded placebo-controlled clinical trial that wasregistered with the website for registration of clinical trials in Iran (http://www.irct.ir: IRCT201610015623N90). Forty women aged 18–40 years with GDM, diagnosed based on the American Diabetes Association guidelines, without prior history of diabetes [20], were included. The study was conducted from July to October 2016. Study protocol was approved by the research ethics committee of Arak University of Medical Sciences (AUMS), and informed consent was taken from all participants. Exclusion criteria were as follows: taking fish oil supplements, insulin therapy, placenta abruption, pre-eclampsia, eclampsia, hypothyroidism, hyperthyroidism, and smokers.

### 2.2. Study Procedures

Subjects were randomly divided into two groups to take either 1000 mg fish oil capsules, containing 180 mg eicosapentaenoic acid (EPA) and 120 mg docosahexaenoic acid (DHA) (*n* = 20) or placebo (*n* = 20) twice a day for 6 weeks. Randomization process was conducted using computer-generated random numbers by a trained staff at the clinic, blinded to both participants and researchers. Omega-3 and placebo capsules were produced by Barij Essence Pharmaceutical Company, Kashan, Iran, approved by Food and Drug Administration (FDA). They were completely identical in terms of their appearance, color, shape, size, smell, and taste and packaging.

To increase compliance rate, all participants received reminder messages on their cell phones every day to remind them to take their capsules. Compliance was evaluated by counting the remaining supplements and subtracting from the number of supplements provided to the participants. Subjects were requested not to change their routine physical activity or usual dietary intakes throughout the study and not to consume any supplements other than the one provided to them by the investigators, as well as not to take any medications that might affect the outcomes during the 6-week intervention. A 3-day food record was obtained at weeks 0, 3, 5, and 6 of the intervention, and macro- and micro-nutrients intakes were determined using the Modified Nutritionist-4 software program (First Databank, San Bruno, CA, USA). Physical activity was described as metabolic equivalents (METs) in hours per day [21].

### 2.3. Assessment of Anthropometric Measures

Weight and height of participants were measured following an overnight fast, using a standard scale (Seca, Hamburg, Germany) at baseline and after 6-weeks’ treatment. BMI was calculated as weight in kg divided by height in meters squared. Weight and length of all newborns were measured in labor ward following the birth by a trained midwife by the use of standard methods (Seca 155 Scale, Hamburg, Germany). Infants’ head circumference was calculated to the nearest 1 mm with a Seca girth measuring tape. We also determined infants’ 1- and 5-min Apgar score as another measure of pregnancy outcome.

### 2.4. Primary and Secondary Outcomes

In this study, PPAR-γ gene expression was considered as the primary outcome, and gene expressions of the low-density lipoprotein receptor (LDLR), interleukin-1 (IL-1), interleukin-8 (IL-8), and TNF-α were considered as the secondary outcomes. In the current study, we quantified gene expression levels related to insulin, lipid, and inflammation in peripheral blood mononuclear cells (PBMCs) from GDM women. PBMCs from venous blood samples are the most available tissue for analysis of gene expression [22]. In addition, gene expression levels related to insulin action, lipid, and inflammation in PBMCs are more accurate than plasma concentrations [22]. Few studies have previously assessed gene expression levels of insulin, lipid, and inflammation in PBMCs from diabetic patients [23,24].

### 2.5. Laboratory Procedures

#### Isolation of PBMCs

At baseline and after 6-week intervention, 10 mL fasting blood samples were taken at Arak reference laboratory. Then, PBMCs were isolated from blood samples of participants with GDM using 50% percoll (Sigma-Aldrich, Dorset, UK). Samples were taken for cell count and viability testing by trypan blue, RNA, and DNA extraction [25].

### 2.6. RNA Extraction and Real-Time PCR (RT-PCR)

RNX-plus kit (Cinnacolon, Tehran, Iran) was used to do RNA extraction. RNA suspension was frozen in −20 °C until it was converted to cDNA. Following the extraction of the total RNAs from each sample, RNA quantification were performed using UV spectrophotometer. Each sample OD 260/280 ratio was intended between 1.7 and 2.1, demonstrating no contamination with both protein and DNA [25]. The isolated RNA was reverse transcribed to cDNA library, using moloney murine leukemia virus (MMLV) reverse transcriptase (RT). Gene expression of PPAR-γ, LDLR, IL-1, IL-8, and TNF-α were evaluated by quantitative RT-PCR in PBMCs, using the LightCycler technology (Roche Diagnostics, Rotkreuz, Switzerland) with SYBR green detection and Amplicon Kit (Table 1). Glyceraldehyde-3-phosphate dehydrogenase (GAPDH) primers were used as housekeeping gene. To design primers, Primer Express Software (Applied Biosystems, Foster City, CA, USA) and Beacon designer software (Takaposizt, Tehran, Iran) were used. Relative transcription levels were calculated using the method of Pffafi or 2^−∆∆*C*T^.

### 2.7. Biochemical Assessment

Fasting plasma glucose (FPG) concentrations were measured on the day of blood collection. Serum insulin concentrations were evaluated by the use of ELISA kit (DiaMetra, Milano, Italy) with inter- and intra-assay coefficient variances (CVs) of 3.4 to 4.7%, respectively. The homeostasis model of assessment-insulin resistance (HOMA-IR) was determined according to the standard formula [26]. Enzymatic kits (Pars Azmun, Tehran, Iran) were used to evaluate FPG, serum triglycerides, total-, and LDL- and HDL-cholesterol levels with inter-and intra-assay CVs lower than 5%. Serum high-sensitivity C-reactive protein (hs-CRP) concentrations were determined by an ELISA kit (LDN, Nordhorn, Germany) with inter- and intra-assay CVs lower than 7%, respectively.

### 2.8. Clinical Assessment

Polyhydramnios was diagnosed using the sonographic estimation method at post-intervention. On the basis of this measurement, polyhydramnios was defined as an amniotic fluid index in excess of 24 cm [27]. Preterm delivery was defined as delivery occurred at <37 weeks of pregnancy and newborn’s macrosomia was defined as birth weight of >4000 g.

### 2.9. Statistical Analysis

The normality of study variables was determined using the Kolmogorov-Smirnov test. Outcome log-transformation was used if model residual has non-normal distribution (PPAR-γ, LDLR, IL-1 and TNF-α). Anthropometric measures, as well as macro-and micro-nutrient dietary intakes, were compared between the two groups, using independent samples *t*-test. The effects of omega-3 supplementation on gene expression related to insulin, lipid, and inflammation, and metabolic profiles, were assessed using independent samples *t*-test. Differences in proportions were evaluated by Fisher’s exact test. The *P*-value of <0.05 was considered statistically significant. All statistical analyses in this study were conducted using the Statistical Package for Social Science version 18 (SPSS Inc., Chicago, IL, USA).

## 3. Results

**Figure 1:**
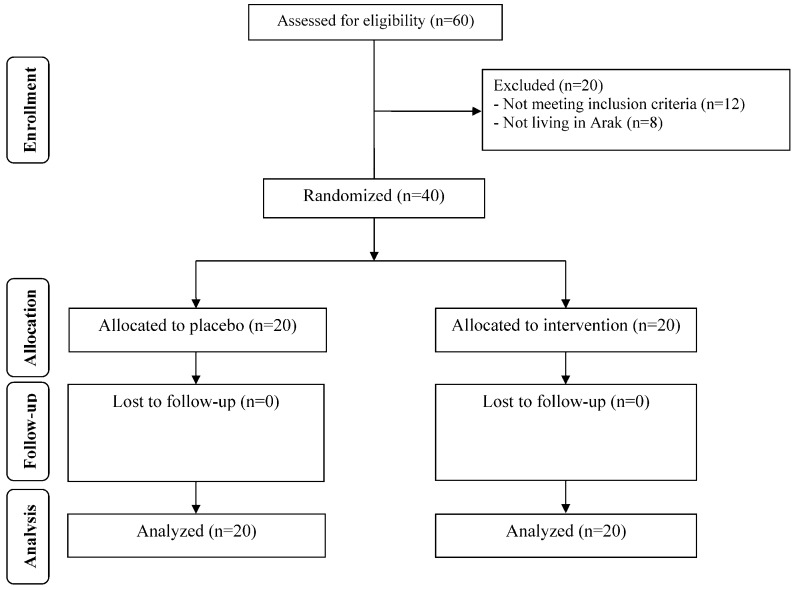
Summary of patient flow diagram.

At the beginning of the study, 60 patients with GDM were invited to enter the trial; however, 20 participants did not meet the inclusion criteria and were excluded from the study. Finally, 40 participants (placebo (*n* = 20) and omega-3 (*n* = 20)) completed the trial (Figure 1). Overall, the compliance rate was high, because more than 90% of capsules were consumed throughout the study in both groups.

Mean age, height, weight, BMI, and METs at baseline and after the 6-week treatment were not statistically different between omega 3 and placebo groups (Table 2).

The mean dietary macro-and micro-nutrient intakes at both baseline and the end of the trial, as well as throughout the intervention, were not significantly different between the two groups (Data not shown).

After the 6-week intervention, compared with the placebo, omega-3 supplementation led to a significant reduction in FPG (−4.4 ± 2.3 vs. +2.9 ± 14.3 mg/dL, *P* = 0.02) and serum triglycerides (−8.3 ± 28.3 vs. +15.7 ± 29.9 mg/dL, *P* = 0.01), and a significant increase in LDL- (+11.2 ± 13.5 vs. +1.6 ± 15.2 mg/dL, *P* = 0.04) and HDL-cholesterol levels (+2.9 ± 3.9 vs. −0.7 ± 5.8 mg/dL, *P* = 0.02) (Table 3). In addition, taking omega-3 supplements was associated with a significant reduction in hs-CRP (−3375.7 ± 4836.8 vs. +82.8 ± 3149.7 ng/mL, *P* = 0.01) compared with the placebo. Omega-3 supplementation did not affect serum insulin, total cholesterol levels, and HOMA-IR compared with the placebo.

We did not find a significant difference in polyhydramnios, gestational age, newborn’s birth size, or Apgar scores when comparing the two groups (Table 4).

**Figure 2:**
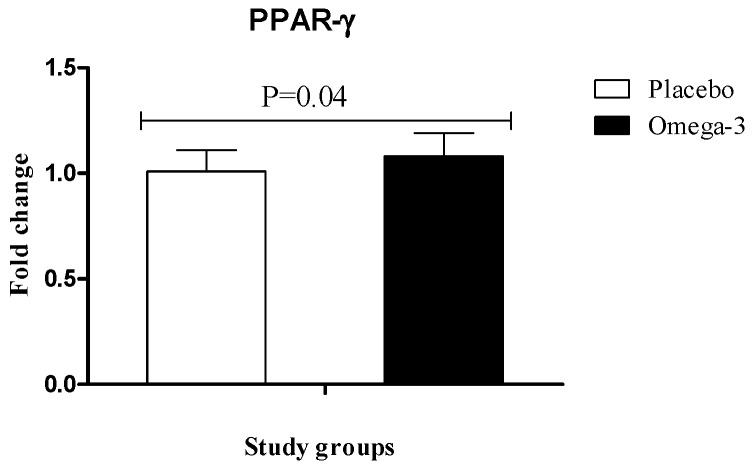
Effect of 6-week supplementation with omega-3 or placebo on expression ratio of PPAR-γ gene in PBMCs of GDM women.

**Figure 3:**
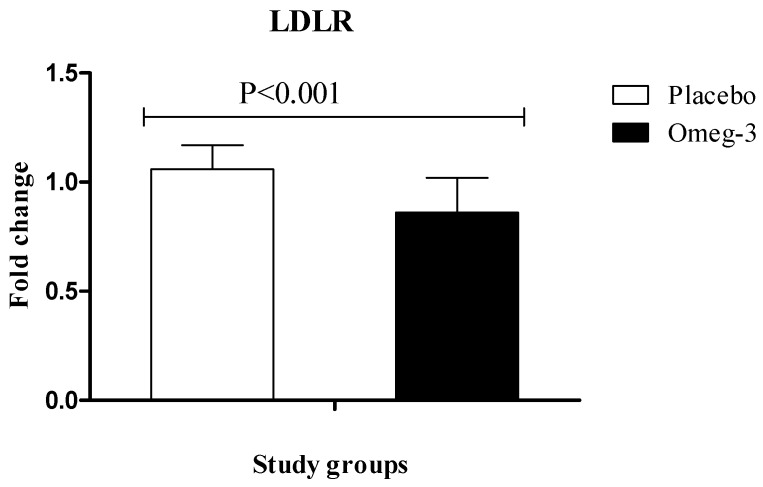
Effect of 6-week supplementation with omega-3 or placebo on expression ratio of LDLR gene in PBMCs of GDM women.

RT-PCR quantitative results showed significant upregulation of gene expression of PPAR-γ (*P* = 0.04) in PBMCs of patients with GDM following omega-3 supplementation, rather than placebo (Figure 2). We also found that compared with the placebo, omega-3 administration downregulated gene expression of LDLR (*P* < 0.001) in PBMCs of participants with GDM (Figure 3).

**Figure 4:**
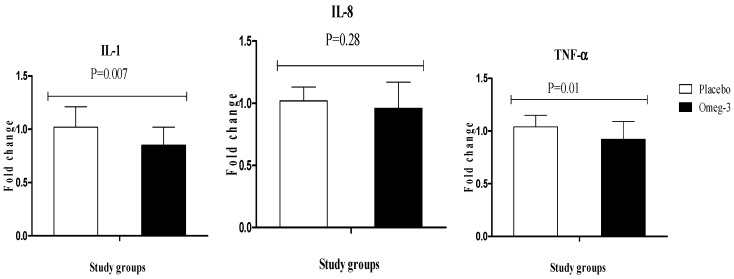
Effect of 6-week supplementation with omega-3 or placebo on expression ratio of IL-1, IL-8, and TNF-α gene in PBMCs of GDM women.

Regarding inflammatory markers, omega-3 supplementation significantly downregulated gene expression of IL-1 (*P* = 0.007) and TNF-α (*P* = 0.01) in PBMCs of patients with GDM; however, it did not affect gene expression of IL-8 (Figure 4).

## 4. Discussion

To our best knowledge, this research is the first study conducted to determine the effect of omega-3 supplementation on gene expression related to insulin action, blood lipids, and inflammation in women with gestational diabetes. We found that omega-3 supplementation for 6 weeks had beneficial effects on gene expression related to improving insulin function, and attenuating lipid and inflammation markers among women with GDM.

The current study indicated that omega-3 supplementation for 6 weeks upregulated PPAR-γ expression and downregulated LDLR expression in PBMCs of women with GDM. In addition, omega-3 supplementation significantly decreased FPG and triglycerides, and significantly increased LDL- and HDL-cholesterol levels, but did not affect serum insulin, total cholesterol levels, and HOMA-IR. In agreement with our study, Anderson et al. [28] showed that supplementation with 3.4 g/day EPA and DHA for 2–3 weeks before having elective cardiac surgery upregulated gene expression of PPAR-γ. In addition, omega-3 intake has been shown to increase gene expression of adiponectin in cultured human adipocytes and mice adipocyte cell lines through affecting the transcription factor PPAR-γ [29,30]. A significant improvement in gene expression of PPAR-γ in pup mice’s brain was also seen following the administration of omega-3 [31].

Omega-3 supplementation might decrease the chance of atherosclerosis and coronary artery disease by improving lipid profiles. Gajos et al. [32] found that omega-3 supplementation reduced Ox-LDL significantly among patients with stable angina. We have previously shown that 1000 mg flaxseed oil and 400 IU vitamin E co-supplementation for 12 weeks decreased gene expression of Ox-LDL in PCOS patients [33]. In hyperlipidemic patients also, EPA intake of 1800 mg/day for 4 weeks significantly decreased Ox-LDL [34]. Among pregnant women, omega-3 supplementation, during pregnancy until three months after delivery, was shown to significantly reduce plasma lipid levels [35], though in another study, Barden et al. [36] did not report any beneficial effect of omega-3 supplementation on maternal lipid profiles.

However, data on omega-3 supplementation and gene expression in women with gestational diabetes are scarce, though current evidence in other study populations is conflicting. Omega-3 supplementation in obese adolescents was shown to be associated with a downregulation of the gene expression of PPAR-γ in adipose tissues [37]. In another study by Nestel et al. [38], omega-3 supplementation for 4 weeks increased Ox-LDL in obese persons. PPARs mainly regulate cell differentiation, glucose, and insulin metabolism, and lipid metabolism. They play an important role in maintaining the metabolic homeostasis [39] and have been associated with lower risk of GDM. Kuzmicki et al. [40] showed lower gene expression of PPAR-γ in patients with GDM rather than those with normal glucose tolerance. In another study, polymorphisms in PPAR-γ were highly associated with GDM occurrence in pregnant women [41]. The mechanisms of PPAR-γ and LDLR gene expression regulation mediated by omega-3 remain mostly unknown. Since fatty acids are the natural ligands of PPAR-γ, omega-3 fatty acids might be able to activate PPAR-γ production [42,43]. Decreased LDLR due to affecting PKB pathway and inhibiting phosphatidylinosinitol 3-kinase and [18] and modifying the activities of oxidative stress-induced NF-κB pathway [44] might be one of the involved mechanisms mediated by omega 3 supplementation.

The current study demonstrated that omega-3 administration for 6 weeks in women with GDM downregulated IL-1 and TNF-α gene expression in PBMCs. In agreement with our findings, anti-inflammatory effects of omega-3 have been previously shown in several studies. Tayyebi-Khosroshahi et al. [45] depicted a significant reduction in TNF-α levels following omega-3 intake in patients on hemodialysis. Ellulu et al. [46] showed beneficial effects of omega-3 supplementation on inflammation and metabolic dysregulation in diabetic patients. Among women with GDM, Samimi et al. [14] did not find any significant change in serum insulin levels and HOMA-IR following omega-3 supplementation; however, in these patients a significant reduction in inflammatory markers was evident. Consistently, Haghiac et al. [47] supplemented pregnant women with omega-3 for more than 6 months and found significant reduction in inflammatory markers. Incorporation of omega-3 into articular cartilage chondrocyte membranes has been shown to significantly decrease gene expression of IL-1α and TNF-α [48]. Fish oil supplementation significantly reduced the gene expression of inflammatory markers in adipose tissue [49]. However, a 8-week fish oil supplementation at a dosage of 3000 mg/day to chronic ambulatory peritoneal dialysis patients did not affect inflammatory markers [50]. Alternatively, a 6-week omega-3 supplementation trial could not affect gene expression of TNF-α and interleukin-6 (IL-6) in PBMCs [51]. Women with gestational diabetes have higher concentration of inflammatory cytokines including IL-6, IL-10, and TNF-α compared with healthy pregnant women [52]. Among women with GDM, higher amounts of TNF-α are secreted by placenta and adipose tissues in response to high glucose and hyperinsulinemia [53]. On the other hand, TNF-α was inversely associated with insulin sensitivity [54]. Omega-3 intake might decrease inflammatory cytokine production by inhibiting the activation of NF-кB [55]. Moreover, it might increase gene expression of PPAR-γ [56], which in turn reduces the production of inflammatory markers.

The main limitation of this study was that we did not evaluate fatty acids profiles levels and plasma adiponectin levels at the baseline and end of the trial. In addition, we could not assess other gene expression related to insulin and lipid. PPAR-γ is a nuclear receptor that requires ligand binding for its activation and subsequent nuclear translocation. Compared to its expression levels, its activation status might be more important. Although the evaluation of expression levels of some downstream target genes of PPAR-γ is interesting, its performance is suggested in next studies. Furthermore, we were unable to administer omeg-3 supplementation for more than 6 weeks due to the particular condition of pregnant women. Although the duration of the intervention was 6 weeks, we followed GDM women until delivery.

## 5. Conclusions

Overall, omega-3 supplementation for 6 weeks in women with gestational diabetes significantly improved gene expression of PPAR-γ, IL-1, and TNF-α; however, it did not affect gene expression of IL-8. In addition, omega-3 supplementation significantly decreased FPG and triglycerides, and significantly increased LDL- and HDL-cholesterol levels, but did not affect serum insulin, total cholesterol levels, and HOMA-IR.

## Figures and Tables

**Table 1:** Specific primers used for real-time quantitative PCR.

Gene	Primer	Product Size (bp)	Annealing Temperature (C)
GAPDH	F: AAGCTCATTTCCTGGTATGACAACG	126	61.3
	R: TCTTCCTCTTGTGCTCTTGCTGG		
PPAR-γ	F: ATGACAGACCTCAGACAGATTG	210	54
	R: AATGTTGGCAGTGGCTCAG		
LDLR	F: ACTTACGGACAGACAGACAG	223	57
	R: GGCCACACATCCCATGATTC		
IL-1	F: GCTTCTCTCTGGTCCTTGG	174	56
	R: AGGGCAGGGTAGAGAAGAG		
IL-8	F: GCAGAGGGTTGTGGAGAAGT	150	56
	R: ACCCTACAACAGACCCACAC		
TNF-α	F: GTCAACCTCCTCTCTGCCAT	188	52
	R: CCAAAGTAGACCTGCCCAGA		

GAPDH, glyceraldehyde-3-Phosphate dehydrogenase; IL-1, interleukin-1; IL-8, interleukin-8; LDLR, low-density lipoprotein receptor; PPAR-γ, peroxisome proliferator-activated receptor gamma; TNF-α, tumor necrosis factor alpha.

**Table 2:** General characteristics of study participants.

	Placebo Group (*n* = 20)	Omega-3 Group (*n* = 20)	*P* ^1^
Gestational age before intervention (weeks)	25.3 ± 1.1	25.4 ± 1.2	0.89
Age (y)	30.8 ± 2.4	30.5 ± 3.8	0.80
Height (cm)	162.1 ± 5.8	161.6 ± 3.4	0.71
Weight at study baseline (kg)	70.6 ± 5.7	73.1 ± 6.7	0.22
Weight at end-of-trial (kg)	72.5 ± 5.7	75.1 ± 6.6	0.19
Weight change (kg)	1.9 ± 0.6	2.0 ± 0.6	0.51
BMI at study baseline (kg/m^2^)	27.0 ± 3.1	28.0 ± 2.6	0.27
BMI at end-of-trial (kg/m^2^)	27.7 ± 3.2	28.8 ± 2.6	0.25
BMI change (kg/m^2^)	0.7 ± 0.2	0.8 ± 0.2	0.54
MET-h/day at study baseline	27.6 ± 2.0	27.1 ± 2.0	0.40
MET-h/day at end-of-trial	27.3 ± 2.1	26.9 ± 2.2	0.46
MET-h/day change	−0.3 ± 0.5	−0.2 ± 0.4	0.73

Data are means ±SDs. ^1^ Obtained from independent *t*-test. METs, metabolic equivalents.

**Table 3:** Metabolic profiles at baseline and 6 weeks after the intervention in patients with gestational diabetes mellitus.

	Placebo Group (*n* = 20)			Omega-3 Group (*n* = 20)			*P* ^1^
	Wk0	Wk6	Change	Wk0	Wk6	Change	
FPG (mg/dL)	95.5 ± 10.1	98.3 ± 10.0	2.9 ± 14.3	95.6 ± 4.3	91.1 ± 3.8	−4.4 ± 2.3	0.02
Insulin (μIU/mL)	12.6 ± 4.7	13.0 ± 6.1	0.4 ± 5.5	13.0 ± 5.7	12.0 ± 4.6	−1.0 ± 8.0	0.51
HOMA-IR	3.0 ± 1.2	3.2 ± 1.6	0.2 ± 1.6	3.1 ± 1.4	2.7 ± 1.0	−0.4 ± 1.9	0.29
Triglycerides (mg/dL)	200.1 ± 56.0	215.8 ± 56.1	15.7 ± 29.9	221.3 ± 80.7	213.1 ± 71.9	−8.3 ± 28.3	0.01
Total cholesterol (mg/dL)	209.5 ± 43.6	213.6 ± 41.5	4.1 ± 19.5	221.7 ± 43.4	234.1 ± 39.2	12.3 ± 15.5	0.14
LDL-cholesterol (mg/dL)	110.3 ± 32.9	1191.9 ± 35.4	1.6 ± 15.2	119.9 ± 34.2	131.1 ± 33.6	11.2 ± 13.5	0.04
HDL-cholesterol (mg/dL)	59.2 ± 15.6	58.5 ± 13.2	−0.7 ± 5.8	57.6 ± 9.1	60.4 ± 9.6	2.9 ± 3.9	0.02
hs-CRP (ng/mL)	6592.2 ± 4476.75	6675.1 ± 4354.4	82.8 ± 3149.7	6911.2 ± 5249.3	3535.6 ± 4529.7	−3375.7 ± 4836.8	0.01

All values are means ±SDs. Obtained from independent *t*-test. FPG, fasting plasma glucose; HOMA-IR, homeostasis model of assessment-estimated insulin resistance; hs-CRP, high-sensitivity C-reactive protein.

**Table 4:** The association of omega-3 supplementation with pregnancy outcomes.

	Placebo Group (*n* = 20)	Omega-3 Group (*n* = 20)	*P* ^1^
Preterm delivery (%)	1 (5.0)	0 (0.0)	0.31 ^†^
Pre-eclampsia (%)	2 (10.0)	1 (5.0)	>0.999 ^†^
Polyhydramnios (%)	2 (10.0)	1 (5.0)	>0.999 ^†^
Macrosomia > 4000 g (%)	3 (15.0)	1 (5.0)	0.60 ^†^
Gestational age (weeks)	39.0 ± 1.2	38.3 ± 1.3	0.11
Newborns’ weight (g)	3337.0 ± 483.7	3467.5 ± 420.3	0.36
Newborns’ length (cm)	51.0 ± 1.7	51.7 ± 2.4	0.27
Newborns’ head circumference (cm)	36.0 ± 1.4	35.7 ± 0.7	0.28
1-min Apgar score	8.7 ± 0.5	8.6 ± 0.5	0.75
5-min Apgar score	9.7 ± 0.5	9.6 ± 0.5	0.75

Values are means ±SDs for continuous measures and are number (%) for dichotomous variables. ^1^ Obtained from independent *t*-test. ^†^ Obtained from Fisher’s exact test.
